# A random effect multiplicative heteroscedastic model for bacterial growth

**DOI:** 10.1186/1471-2105-11-77

**Published:** 2010-02-08

**Authors:** Ricardo Cao, Mario Francisco-Fernández, Emiliano J Quinto

**Affiliations:** 1 Department of Mathematics, University of A Coruña, School of Computer Science, Campus de Elviña, s/n, 15071 A Coruña, Spain; 2 Department of Food Science and Nutrition, University of Valladolid, School of Medicine and Health Sciences, Avenida Ramón y Cajal 7, 47005 Valladolid, Spain

## Abstract

**Background:**

Predictive microbiology develops mathematical models that can predict the growth rate of a microorganism population under a set of environmental conditions. Many primary growth models have been proposed. However, when primary models are applied to bacterial growth curves, the biological variability is reduced to a single curve defined by some kinetic parameters (lag time and growth rate), and sometimes the models give poor fits in some regions of the curve. The development of a prediction band (from a set of bacterial growth curves) using non-parametric and bootstrap methods permits to overcome that problem and include the biological variability of the microorganism into the modelling process.

**Results:**

Absorbance data from *Listeria monocytogenes *cultured at 22, 26, 38, and 42°C were selected under different environmental conditions of pH (4.5, 5.5, 6.5, and 7.4) and percentage of NaCl (2.5, 3.5, 4.5, and 5.5). Transformation of absorbance data to viable count data was carried out. A random effect multiplicative heteroscedastic model was considered to explain the dynamics of bacterial growth. The concept of a prediction band for microbial growth is proposed. The bootstrap method was used to obtain resamples from this model. An iterative procedure is proposed to overcome the computer intensive task of calculating simultaneous prediction intervals, along time, for bacterial growth. The bands were narrower below the inflection point (0-8 h at 22°C, and 0-5.5 h at 42°C), and wider to the right of it (from 9 h onwards at 22°C, and from 7 h onwards at 42°C). A wider band was observed at 42°C than at 22°C when the curves reach their upper asymptote. Similar bands have been obtained for 26 and 38°C.

**Conclusions:**

The combination of nonparametric models and bootstrap techniques results in a good procedure to obtain reliable prediction bands in this context. Moreover, the new iterative algorithm proposed in this paper allows one to achieve exactly the prefixed coverage probability for the prediction band. The microbial growth bands reflect the influence of the different environmental conditions on the microorganism behaviour, helping in the interpretation of the biological meaning of the growth curves obtained experimentally.

## Background

A primary objective in food microbiology is to identify, quantify, and know the behaviour of foodborne microorganisms. However, the inherent inaccuracies in the enumeration process and the natural variation found in all bacteria populations complicate these tasks [1]. In the 1980s, the increase in the incidence of foodborne outbreaks led to a major demand of a safe food supply. At the same time many microbiologists were beginning to accept that traditional microbiological methods to determine food quality and safety were limited by the time needed to obtain results. An alternative is predictive microbiology, which relies upon the development of mathematical models that can predict the growth or decline rates of microorganisms under a given set of environmental conditions [2]. In a general sense, a model simplifies a system by using a combination of descriptions, mathematical functions or equations, and specific starting conditions. There are two general classes of models in this context: descriptive and explanatory [1]. Descriptive, observational, or empirical models are data-driven, and it is difficult to make true predictions from them because they cannot be extrapolated beyond the data used to build them. Explanatory, or mechanistic models aim to relate the given data to fundamental scientific principles. Many predictive microbiology models have parameters that are related to observed phenomena. Excellent reviews and extended discussions about the potential benefits of predictive models can be found in the literature [1-4]. Many growth models have been proposed since 1980. The classical sigmoid growth functions, especially the modified logistic and Gompertz equations, must be mentioned [5,6]. Over the last decade, a new generation of models have been developed: Baranyi model [3,7], Hills model [8,9], Buchanan model [10], and the heterogeneous population model [11].

Current available approaches (i) reduce the bacterial growth variability to a single curve, and (ii) sometimes, under different environmental conditions, the models offer poor fits in some regions of the growth curve. Instead of using standard primary models, the method presented in this paper relies on nonparametric estimation of the trend of the growth curve that incorporates random fluctuation in time as well as biological variability of microorganisms. Nonparametric methods do not assume a prespecified functional form (as linear, quadratic or logistic) for the viable count or absorbance. The predictions based on these methods are model-free, in the sense that there is no need to build a different mathematical model for each specific setup. A method for constructing simultaneous prediction bands (not just pointwise confidence or prediction intervals) for the bacterial growth is proposed. This method accounts for random fluctuation in time as well as for biological variability. This is done without imposing inflexible parametric restrictions used in parametric models. The method lets the data speak by themselves. It provides a nonparametric estimation of the best-fit line and nonparametric prediction bands, constructed using the bootstrap. These bands are designed to contain all the points of a future growth curve with a prescribed high probability, typically 95%. Nevertheless, the interest here is on bacterial growth prediction, rather than in bacterial growth modelling. On the other hand the traditional effect of secondary models could be incorporated into the proposed method via some extension of it.

It is not the purpose of this paper to model the growth of a microorganism along a wide range of environmental conditions. That work can be done using available databases. The objective is to propose and apply a new model to any sigmoid curve (e.g., viable count data, absorbance data, etc.). To our knowledge, the procedure proposed in this paper has not been considered before for these purposes. Schaffner [12] used a statistical bootstrapping technique (see [13]) to simulate growth rate measurements from a single set of experiments, with the objective of estimating their variance. Oscar [14-16] used a prediction interval to model the variation of the growth of *Salmonella *in different chicken samples. Within this context, the aim of the present study was to apply a random effect multiplicative heteroscedastic model to show its behaviour with absorbance or viable count data, and also to explain the dynamics of bacterial growth of *Listeria monocytogenes *under different conditions of temperature, pH, and NaCl. The bootstrap method and an iterative procedure are also proposed. The concept of a prediction band for microbial growth is used.

## Methods

### Microorganism and inoculum preparation

The strain used in this study was a *Listeria monocytogenes *strain previously isolated from poultry meat (Department of Animal and Food Sciences, School of Veterinary Medicine, Autonomous University of Barcelona, 08193, Bellaterra, Barcelona, Spain). The strain was reconstituted in Brain Heart Infusion (BHI, Difco Laboratories, Detroit, Mich., USA) and incubated at 37°C for 24 h.

### Determination of growth curves and linear range

Bottles with 250 ml of BHI were prepared. Combinations of different values of pH and percentages of NaCl were considered. The pH value was adjusted in each bottle to 4.5, 5.5, 6.5 and 7.4 with HCl and NaOH. The percentage of NaCl was adjusted to 2.5, 3.5, 4.5, or 5.5. Nine ml from each bottle were transferred to tubes and sterilized by autoclaving at 121°C for 15 min. These tubes were used to dilute the inoculum of the microorganism previously activated in BHI at 37°C for 24 h (ca. 4.8 × 10^9 ^CFU/ml). Two hundred *μ*l from dilution 10^-3 ^(ca. 4.8 × 10^6 ^CFU/ml) were distributed into 96-well micro-titer plates and immediately incubated at 22, 26, 38, or 42°C on a microplate reader (SLT 340 ATTC, SLT Labinstruments, Austria) for 15-24 h. Twenty replications of each combination of temperature, pH, and NaCl were done. Two hundred *μ*l of BHI as control test was also distributed into the microplate wells and incubated at the same conditions. The absorbance measurements were done at a wavelength of 595 nm and taken every 15 min. The cultures were inoculated from the stationary phase because more reproducible results can be obtained than from the log phase [17].

The linear range was determined by plotting absorbance vs. CFU/ml. The population of *L. monocytogenes *from BHI cultures was enumerated in PCA (Difco) at 31°C for 24 h (ca. 4.8 × 10^9 ^CFU/ml), and it was diluted in BHI at 1/2, 1/4, 1/5, 1/8, 1/10, 1/16, 1/20, 1/50, 1/100, 1/500, and 1/1000. Aliquots of 200 *μ*l from each bacterial dilution were inoculated into 6 wells of the 96-well microtiter plates to measure their absorbance. Non-inoculated BHI was placed in 12 wells of the same microtiter plate. Absorbance was read in the microplate reader (SLT 340 ATTC, SLT Labinstruments, Austria) at 595 nm. The threshold of detection, corresponding to the bacterial concentration that involves a significant change of the absorbance, was observed when the measured values exceeded 0.111, which corresponded to ca. 4.8 × 10^7 ^CFU/ml. This value was in the linear range of the calibration curve.

### Statistical methods

In view of the shapes of the bacterial growth curves, an experimental design model has been considered. Some simple method for parameter estimation has been used. A bootstrap resampling plan has been designed in order to construct simultaneous prediction intervals for the bacterial growth curves.

#### Random effect multiplicative model

The following random effect multiplicative heteroscedastic linear model has been considered:(1)

where *I *is the number of wells, *J *is the number of sampled instants along the time range and *Y*_*ij *_is the absorbance for well *i *at time *j*. The basic assumptions for this model are the following. The terms of the well component, *α*_*i*_, account for a random fluctuation factor, with mean 1. The actual value of *α*_*i *_accounts for an overfitting or an underfitting of the mean absorbance curve along time. Its variance is  and its distribution is assumed to be normal. The time mean effects, *μ*_*j*_, are unknown values that model the overall well mean absorbance along time. The positive constants  are the absorbance variances for the *j*-th time instant and the errors, *ε*_*ij*_, are standard normal random variables that account for experimental error. As a consequence the *Y*_*ij *_are normally distributed with mean *μ*_*j *_and variance . Model (1) is an extension of a principal mixed effect model (see [18], for instance).

#### Parameter estimation

A very simple approach has been adopted for parameter estimation. The method of moments has been used to obtain estimators for the mean (*μ*_*j*_) and the variance () time effect, as well as for the variance of the well random effect ():

where • denotes average along the pertaining index,

In practice, outliers may seriously affect the estimators  and  above. For this reason, robust versions of these have been used:  and , where *MAD*(*x*_•_) = Median (|*x*_*i *_- *Me*|) and *Me *= Median (*x*_•_) and Φ is the standard normal cumulative distribution function. These robust versions are based on the fact that, for a normal distribution with mean *μ *and standard deviation *σ*, the following relationship, between its dispersion and its *MAD*, holds: *σ *= *MAD*·Φ^-1 ^(0.75).

#### Boostrap resampling plan

In order to construct simultaneous prediction intervals a bootstrap resampling method (see [13]) has been considered to mimic the joint probability distribution of the random vector (*Y*_*i*1_, *Y*_*i*2_,..., *Y*_*iJ*_). To that aim, the following procedure has been designed:

1. Given the original absorbance sample, *Y*_*ij *_(*i *= 1, 2,..., *I*, *j *= 1, 2,..., *J*), compute the estimations ,  (*j *= 1, 2,..., *J*) and  detailed in the previous subsection.

2. Fix the number of bootstrap resamples, *B*, typically a large number (*B *= 1000 or 5000, for example).

3. For every *b *= 1, 2,..., *B*, draw bootstrap random well effect replications, , from a normal distribution with mean 1 and variance , and the bootstrap version of the experimental error, , (*j *= 1, 2,..., *J*) from a standard normal distribution.

4. Using the bootstrap analogue of the well effect (), the bootstrap random errors () and the estimators from the original sample (, ), the bootstrap version of the absorbance is easily defined via (1):

The sample of simulated vectors  (*b *= 1, 2,..., *B*) can be used to approximate the joint distribution of the random vector (*Y*_*i*1_, *Y*_*i*2_,..., *Y*_*iJ*_), which is needed to construct the prediction band.

#### Bootstrap prediction band

Since the number of sampled time instants is usually moderate or high, correction for multiple prediction intervals is an important issue. Given an initial prediction level, 1 - *α*, for a small *α *(*α *= 0.01 or 0.05, typically), marginal (1 - *α*)-prediction intervals, (ℓ_*j*_, *u*_*j*_), for every time instant *j *= 1, 2,..., *J *can be easily constructed. Their endpoints, ℓ_*j *_and *u*_*j*_, are the th and th ordered statistics of the resample , where ⌈*x*⌉ denotes the integer part of *x*. In other terms, ℓ_*j *_and *u*_*j*_, are the values that are in positions  and , when sorting the bootstrap resample in an increasing order.

Individual prediction intervals have approximately the nominal coverage probability (1 - *α*) when they are considered separately (for a particular sampled instant). However, the probability that the whole growth curve is included in the band depicted by the whole set of intervals is much smaller. This is known as the multiple range testing problem (see [19]) or the false discovery rate in high dimensional statistical problems (see [20]).

A classical way to correct for multiple testing is the popular Bonferroni approach (see [21]). In a hypothesis testing context, the idea behind this approach is to consider a new significance level, , and compute individual tests using this new level. The resulting multiple test has a multiple level which is much closer to the desired *α*. However, it is well known that the Bonferroni approach is a conservative procedure. In our context, this means that the joint coverage probability of the prediction band would be larger than the desired 1 - *α*.

Starting from the conservative Bonferroni approach and the anticonservative individual testing approach, the following algorithm finds an approximate (1 - *α*)-prediction interval, with a given approximation error *δ *(typically *δ *is small in comparison with the nominal *α*, for instance ):

1. Fix  and . Fix the iteration number, k = 0.

2. Compute 

3. Use the bootstrap resamples to compute individual predictions intervals with ,  and  prediction levels.

4. Compute with the same bootstrap resamples, the proportion of simulated growth curves that are included in each of these confidence bands. These proportions satisfy ,  and 

5. If , then define  and . Otherwise define  and .

6. Stop at step *k *if . Otherwise increase *k *in one unit and repeat Steps 2-5.

The final approximate (1 - *α*) simultaneous prediction intervals are those obtained for level  in the last iteration.

## Results

Absorbance results from the combination of pH 4.5 and 5.5% of NaCl at all temperatures were eliminated for subsequent calculations, since no growth curve was observed along the study period. In order to show the prediction bands, several scenarios for the temperatures have been considered. Temperatures of 22 and 26°C could be defined as "room temperatures", and 38 and 42°C were selected as "highly abusive temperatures".

**Figure 1:**
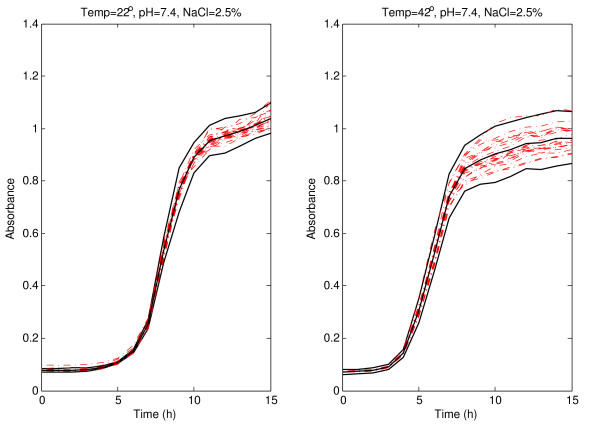
**Prediction band for *Listeria monocytogenes *absorbance growth curves at 22 and 42°C**. Prediction band and best-fit line (solid lines) for *Listeria monocytogenes *absorbance growth curves (dash-dotted lines) at 22 and 42°C with pH 7.4, and 2.5% of NaCl.

**Figure 2:**
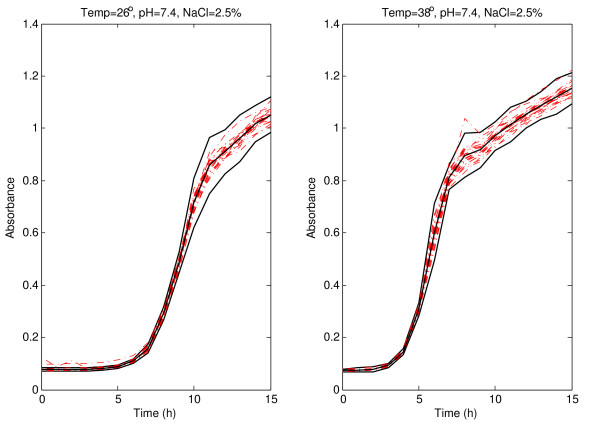
**Prediction band for *Listeria monocytogenes *absorbance growth curves at 26 and 38°C**. Prediction band and best-fit line (solid lines) for *Listeria monocytogenes *absorbance growth curves (dash-dotted lines) at 26 and 38°C with pH 7.4, and 2.5% of NaCl.

The robust version of the prediction bands, presented in the previous Section, for pH 7.4 and 2.5% of NaCl at 22 and 42°C with *α *= 0.05 are shown in Figure 1. Experimental absorbance data obtained under different environmental conditions are shown. As expected with the selected confidence (95%), the inclusion of the vast majority of the absorbance curves within the bootstrap prediction bands was observed. The bands for absorbance sigmoid curves are narrower during the first part of the curves (before the inflection point), that is, between 0 and 8 h at 22°C, and between 0 and 5.5 h at 42°C, approximately; and they are wider to the right of the inflection point: from 9 h onwards at 22°C, and from 7 h onwards at 42°C. A wider band was observed at 42°C than at 22°C over the inflection point. Similar patterns have been obtained for 26 and 38°C (Figure 2). The narrow initial zone of the band is between 0 and 9 h at 26°C and between 0 and 5.5 h at 38°C. From 11 h onwards at 26°C and from 7 h onwards at 38°C, the bands are wider and arrive to the upper horizontal asymptote.

**Figure 3:**
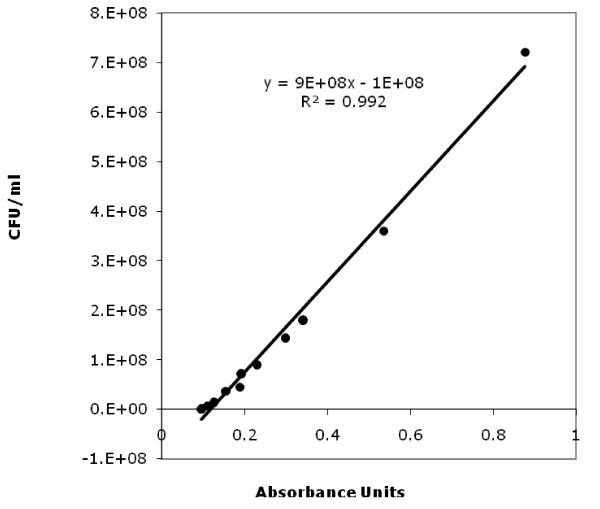
**Linear range of absorbance vs viable counts**. Linear regression fit to explain the relationship between absorbance and viable counts.

Automated measures are commonly used to estimate bacterial growth parameters. Unfortunately, little information is obtained on the lag phase because the change in the physical properties of a culture is detectable only at high cell concentrations [22]. In the present study the new model has been applied to sigmoid absorbance (optical density) curves. However, this technique could be equally applicable to viable count data or any other unit of measure. Thus, showing the applicability of the model, the previously used absorbance data has been transformed into viable counts using the linear range formula (Figure 3). Baranyi and Roberts [23] suggested that the linear calibration function has to be established over a complete matrix of environmental variables. However, a calibration linear range obtained under optimal conditions gives enough information for its application to a complete range of environmental variables. In order to study properly the characteristics of the microorganisms, there is a need to know their general behaviour. To study the correlation between an automatic measure and the viable count every time the conditions change requires a big effort. Following the results of Robinson et al. [17], the cells were initially in a similar physiological state. Therefore, Baranyi and Roberts [3] stated that for cultures having identical physiological states at inoculation and being cultivated under constant (but different) temperatures, the relationship between the lag time and the maximum specific growth rate is maintained. Since the purpose of the present work is to show the prediction bands rather than to study in depth the modelling of the data (a possibility for further studies), a linear relationship was used from the optimal conditions for *L. monocytogenes *at 31°C. The transformation implies that some of the first points from the absorbance curve gives negative values that, obviously, represent points out of the linear range. As it has been stated before, the linear range starts with an absorbance value of 0.111. This value corresponds with the end of the lower horizontal asymptote (detection time or bacterial concentration that involves a significant change in absorbance), so little data has been lost in the transformation from absorbance to viable counts. Those points have not been considered for further calculations. All positive values (viable counts) have been introduced in the model and it has been rerun.

**Figure 4:**
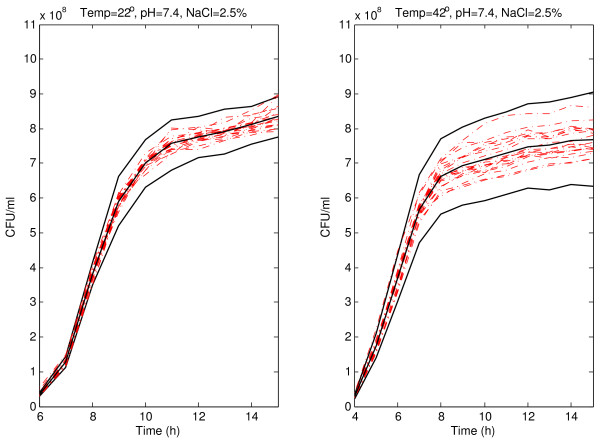
**Prediction band for *Listeria monocytogenes *viable count curves at 22 and 42°C**. Prediction band and best-fit line (solid lines) for *Listeria monocytogenes *viable count curves (dash-dotted lines) at 22 and 42°C with pH 7.4, and 2.5% of NaCl.

**Figure 5:**
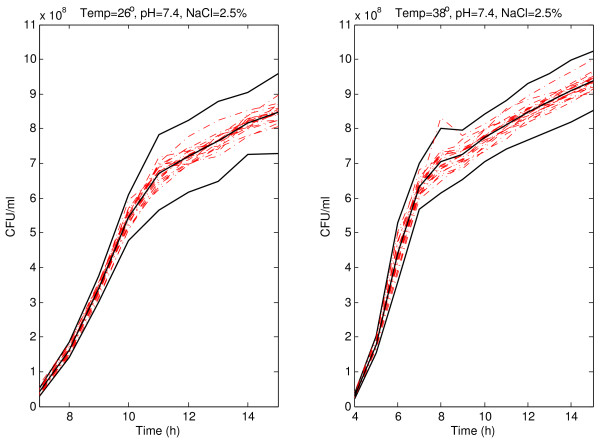
**Prediction band for *Listeria monocytogenes *viable count curves at 26 and 38°C**. Prediction band and best-fit line (solid lines) for *Listeria monocytogenes *viable count curves (dash-dotted lines) at 26 and 38°C with pH 7.4, and 2.5% of NaCl.

The viable count prediction bands for pH 7.4 and 2.5% of NaCl at 22 and 42°C, and for pH 7.4 and 2.5% of NaCl at 26 and 38°C are shown in Figures 4 and 5, respectively. As expected the vast majority of the growth curves were inside the bootstrap prediction bands. The difference between both temperatures 22 and 42°C is very clear because the same band width is observed after 9 h at 22°C and after 7 h at 42°C (at 9 h the band is wider). The variability of viable count curves increases at the end of the experiment. Prediction bands reflect the variability increasing the width in that area. The same situation would be observed during the first part of the curves if the variability was greater. Since the original data come from turbidimetric technique, the variability is lower that it would has been from plate counts. The band width is greater at 42°C suggesting that under less favourable environmental conditions the prediction bands reflect the increasing variability derived from that conditions. Under more favourable conditions (22, 26, or 38°C) the band width is smaller. Obviously the adverse conditions have an influence on the microorganisms, and it is reflected in the absorbance curves and, of course, in the viable count curves. The prediction bands reflect those influences and trends, helping in the interpretation of the biological meaning of the curves obtained experimentally.

## Discussion

No standard primary model has been applied to the experimental data. It is not the aim of this study. Multiple parametric models for *L. monocytogenes *and *L. innocua *in different media under several environmental conditions have been published [24-33]. Moreover, the behaviour of this microorganism under different environmental conditions can be searched with the ComBase (Combined Database for Predictive Microbiology, ComBase Initiative, Institute of Food Research, Norwich Research Park, Colney, Norwich NR4 7UA, UK), a database of microbial responses to food environments. This database was preceded by two independent, but similar initiatives: the Food MicroModel (FMM) (Food MicroModel Ltd., Randals Road, Leatherhead, Surrey, KT22 7RY, UK), and the Pathogen Modeling Program (PMP) [34]. The FMM is no longer in operation, but the PMP can be consulted and downloaded from its web page. It provides the upper and lower confidence limits indicating the variation in the predictions at a confidence level of 95%. On the other hand, well-known curve fitting software tools are the MicroFit software tool (Institute of Food Research, UK) and the DMFit Dynamic Modelling Excel add-in [3]. Moreover, the Seafood Spoilage Predictor (SSP) software [35] includes microbial growth models for seafood products.

It is important to note that the exponential phase of growth curves is included within the prediction band of microbial growth. Lag and exponential phases are the most studied in food microbiology, and the most used in predictive microbiology research. Their biological significance is determined by the time needed by the microorganisms to adapt their metabolism routes to the surrounding media and to start the exponential growth until the maximum population is reached in that environment. So, it is extremely important that these growth phases are included within the prediction band of microbial growth.

Unfortunately, the use of absorbance makes difficult the investigation of the lag time. Moreover, the transformation of absorbance data to viable count data eliminated the first points since they are not in the linear relationship range between optical density and viable counts. However, a good view from the inflection point onwards can be obtained. If a complete viable count curve were introduced in the model, all phases of the growth curve would be obtained and included within the prediction band.

Microbiological data provides little insight into the relationship between physiological processes and growth, and the use of mathematical models is a way to link them. In its simple form a mathematical model is just a mathematical description of a process [1]. Many growth models have been proposed since 1980. They generally differ on how precisely they describe the microorganism growth phases.

Sigmoid models were used to describe the increase of the bacterial cell density vs time. The logistic model and the modified Gompertz model should be mentioned among them [5,6]. These two models were not initially formulated for describing the microbial growth, but they were adapted and reparameterized. Baranyi et al. [7] proposed a less empirical growth model based on a differential equation. A new version of the model was later developed [3].

Hills and Wright [9] proposed a compartmental model. The first compartment describes the evolution of all chromosomal material vs time, and the second compartment describes the evolution of all nonchromosomal material vs time. Buchanan et al. [10] discussed that the transition between the lag phase and the exponential phase is due to the inter-cell variability, and they assumed that this variability is small. They proposed a model with an abrupt transition between those growth phases. McKellar [11] proposed a compartmental model based on the assumption that within a bacterial population freshly inoculated in a rich medium, some cells will grow and some will never grow.

Baranyi [36] published a population-structured model assuming that the bacterial population could be divided into cells still in the lag phase and cells in the exponential phase. The author assumed that cells transform from the lag to the exponential phase at a constant rate. Recently, McKellar and Knight [37] and McKellar [38] developed two other models.

Baty and Delignette-Muller [39] compared some of these models in terms of biological meaning, mathematical definition and statistical fitting properties. López et al. [40] evaluated the suitability of several mathematical functions (three-phase linear, logistic, Gompertz, Von Bertalanffy, Richards, Morgan, Weibull, France and Baranyi) for describing microbial growth curves. Van Impe et al. [41] proposed a novel class of microbial growth models in contrast with the currently used logistic type models. The novel model class explicitly incorporates nutrient exhaustion and/or metabolic waste product effects.

Each microorganism can have different behaviour under the same environmental conditions, but it has different ways to survive depending on (i) the surrounding media and its nutrients, (ii) the number of microorganisms that constitute the initial population, (iii) the communication among the individual members of the population (quorum sensing), and finally, (iv) the probability that the microorganism has to survive alone in a favourable or unfavourable environment.

Primary models do not explicitly incorporate the biological variability. When applied to a set of bacterial curves these models reduce to a single curve defined by some kinetic parameters. If more experiments are carried out under the same environmental conditions, the new curves will never adjust perfectly to the primary model. The different behaviour of each cell of *Listeria monocytogenes *inside a well has its own influence on the growth kinetics of the population. Thus, the growth curve obtained from each well is different because of that biological variability. However, we used nonparametric methods (that are, flexible with every type of curve) and developed a prediction band (from a set of curves) with the property that the 100·(1 - *α*)% of the new curves will be inside this band. Obtaining a prediction band using a primary model has been the aim of this study. The variability has been included in the model (1) with the well random effect and the experimental error. The combination of non-parametric methods with bootstrap techniques has been applied. We used a prefixed coverage of 95%, although different levels could be used. A 95% prediction band means that with a probability of 0.95 a new whole curve (not used to build the model) would be within it. This is acceptable from a microbiological point of view, because the inherent biological variability will never permit the inclusion of all growth curves within a band. Obviously, the narrower the band (precision) and the higher the coverage probability, the better. Unfortunately, those two variables (the coverage and the precision) work in reverse order. That is, if the coverage is increased (e.g., from 95% to 99%), the band is going to be wider (worthless for modelling purposes), and vice versa. 

Provided by the Prism program (GraphPad Software, San Diego, California), Oscar used a 95% prediction interval to model the variation of *Salmonella *Typhimurium DT104 growth in heterogeneous food matrixes: ground chicken breast meat [14], chicken frankfurters [15], and chicken skin [16]. The intervals were used succesfully to capture experimental error, the uncertainty of the curve fit, and the scatter of the growth data around the curve [14-16]. This author developed primary, secondary, and tertiary models for predicting the growth of the microorganism. Model verification and validation was done using 95% prediction intervals. These intervals are construted in order to contain 95% of future CFU data (or points from a experimental growth curve). However, no secondary or tertiary models were used in the present work because it was not the aim of this study. Moreover, the target of our approach is different to that of the prediction intervals computed in those papers. Our prediction band would include 95% of future new experimental growth curves (that is, all the points from such curves).

It is important to note that a prediction band with a coverage probability of (1 - *α*) is not the band obtained by joining the corresponding (1 - *α*) prediction intervals at observed times, because the probability that a new whole curve is within such a band would be smaller that (1 - *α*). There are several methods in the literature trying to correct this problem and to obtain a band with coverage probability (1 - *α*), for example [21]. However, it has been demonstrated that Bonferroni correction does not work properly, because it is too conservative (see [20]). In the present paper, a new iterative technique is proposed to achieve an almost exact (1 - *α*) prediction band. This technique was previously described in detail in the bootstrap prediction band subsection. We performed an experiment to compare our approach with the one using the band obtained by joining the corresponding (1 - *α*) confidence intervals at observed instants. The coverage probabilities in each of the individual intervals and the simultaneous coverage probability for the band have been computed when using pointwise 95% prediction intervals without any further correction. We also computed the simultaneous coverage probability of the bands proposed in this paper with the new method. It is worth noting that these percentages are estimated with low accuracy, since these estimations are only based on 20 observed curves. The conditions are those shown in Figures 1, 2, 4 and 5. It was clearly observed that, although the coverage probabilities of the individual intervals are close to 95%, the simultaneous coverage of the band is drastically reduced if no multiple range correction is performed. However, the prediction bands proposed in this paper exhibit simultaneous coverage probabilities much closer to 95%.

The preceding estimation procedure and bootstrap simultaneous prediction intervals algorithm have been applied to a sample of bacterial growth curves corresponding to *Listeria monocytogenes*, and a prediction band for microbial growth has been computed. *Listeria monocytogenes *has been recognized as an important foodborne pathogen that causes listeriosis. Outbreaks of listeriosis have been associated with milk, cheese, vegetables and salads, and meat products [42]. The microorganism is particularly problematic for the food industry because it is widespread in the environment [43,44]. Foods are heterogeneous systems because of (i) the wide range of types (vegetables, meat, dairy products, etc.) and (ii) their internal differences. A band of microbial growth provides a safer estimation than a curve for the bacterial behaviour under different conditions. This fact could be very useful from the public health point of view. With the application of this technique, the biological variability of the microorganism would be considered in the elaboration of the band, a safer knowledge of the microorganisms' behaviour could be obtained, and the risks of wrong microbial parameters is minimized. The risk increases when the result of this application is an underestimation of the kinetic growth parameters. It has to be also noted that the prediction bands obtained must be only considered for the environmental conditions used in the study. The same argument is applied to other primary models.

From the statistical viewpoint, the normality assumption made on the random effect (*α*_*i*_) and the error (*ε*_*ij*_) can be relaxed using semiparametric models that incorporate adaptive estimation of the parameters using preliminary nonparametric density estimation [45]. This very time consuming approach will be the object of future research.

A Matlab function called REMHM.m, implementing the estimation for the random effect multiplicative heteroscedastic model and the bootstrap algorithm for constructing simultaneous prediction intervals can be found in Additional file 1. A file containing some information about the program REMHM.m is in Additional file 2. Moreover, files with the absorbance data used in this paper can be found in Additional file 3. All these files are also available at the web page http://dm.udc.es/modes/?q=en/node/256. 

Additional effects, as those coming from pH or temperature, could be added directly to the model presented in (1). This would be a hybrid approach incorporating the roles of classical primary and secondary models at a time. Although this approach may be useful for modelling bacterial growth as a function of time, biological variability and environmental variables, it seems not appropriate for prediction bands. When constructing a prediction band the interest is in finding some limits for the growth curve, with high probability, for some particular values of environmental variables, as pH or temperature. This is the reason why this hybrid model is not analyzed in this paper, focused on prediction bands.

For a semiparametric approach, like the one presented in this paper, it is not obvious to define the analogue of the classical parameters used in bacterial growth: inoculum, lag, maximum growth rate and maximum population density. The definition of these values is more or less straightforward in many parametric models like Gompertz, due to the restrictions imposed by the model. Extension of this definitions to nonparametric or semiparametric models like the one presented in this paper will be the object of future research.

## Conclusions

In this article, we proposed a random effect multiplicative heteroscedastic model to explain the dynamics of bacterial growth of *Listeria monocytogenes *under different conditions of temperature, pH, and NaCl. Instead of using standard primary models, based on parametric fits, like the Gompertz model, for example, the method presented in this paper relies on nonparametric estimation of the trend of the growth curve that incorporates random fluctuation in time as well as biological variability of microorganisms. Using a bootstrap resampling method and an iterative algorithm, a procedure for constructing simultaneous prediction bands for the bacterial growth is proposed. The basic steps to construct these bands are the following. (a) Use the observed bacterial data to estimate the unknown parameters in the random effect multiplicative heteroscedastic linear model given in (1). (b) Simulate several thousands of artificial bacterial growth curves (bootstrap resamples) from model (1) with estimated parameters. (c) Use these boostrap resamples to obtain a Monte Carlo approximation of the 95% simultaneous prediction band. Classical approaches in this context reduce the bacterial growth variability to a single curve and sometimes offer poor fits in some regions of the growth curve. The method considered in this paper gives more flexiblity and the prediction bands incorporate the biological variability of the microorganism. The procedure can be applied to any sigmoid curve (e.g., viable count data, absorbance data, etc.) and to different microorganism. A Matlab program implementing this procedure has been developed by the authors.

## Authors' contributions

All authors conceived the project. EJQ carried out the laboratory studies and the preparation of the microbial data. RC and MFF designed the model and the algorithms presented in the manuscript. MFF implemented the algorithms and performed the computational experiments. All authors analyzed the results and were involved in manuscript preparation. All authors read and approved the final document.

## Supplementary Material

Additional file 1 **Program for computing the prediction bands**. Program for computing the prediction bands with the method described in the paper (Matlab software is needed to run this program) Click here for file

Additional file 2 **Information about Matlab program REMHM.m**. File containing some information about the program REMHM.m Click here for file

Additional file 3 **Absorbance and viable count data**. Compress file, containing the data (files Temp22pH74NaCl25.mat, Temp42pH74NaCl25.mat, Temp26pH74NaCl25.mat, Temp38pH74NaCl25.mat, Temp22pH74NaCl25b.mat, Temp42pH74NaCl25b.mat, Temp26pH74NaCl25b.mat, Temp38pH74NaCl25b.mat) to obtain the bands shown in Figures 1, 2, 4 and 5. Click here for file
